# Evolutionary genomics: the fruits of genomic approaches applied to evolutionary biology

**DOI:** 10.1186/s13059-018-1615-x

**Published:** 2019-01-10

**Authors:** Timothy R. Sands

**Affiliations:** Genome Biology, BMC, London, UK

The study of evolution has been transformed in recent years by the use and integration of ‘big data’, permitting new approaches to be applied to tackle longstanding problems, application to an ever-widening range of species as technologies become cheaper and more accessible, and opening up entirely new questions about the evolution of genomes. *Genome Biology* has recently published a special issue on Evolutionary Genomics [1], demonstrating the diversity of research that applies genomic data to the study of evolutionary processes in different species and within the genome itself. The special issue was guest-edited by Sarah Tishkoff from the University of Pennsylvania and Hans Ellegren from Uppsala University.

The increased resolution available from detailed sampling and genome-scale data gives an increasingly clear picture of the patterns and mechanisms of adaptation in many species, from model organisms to less frequently studied species. In this special issue François Mallard and colleagues [2] found that in *Drosophila*, contrary to expectation, large-effect loci at intermediate allele frequencies allow rapid adaptation to thermal selection. Similarly, Jing Wang and colleagues showed that in Aspen, a large effect locus controls the timing of bud set and rapid adaptation to shorter growing seasons and colder climates [3]. Katie Lotterhos and colleagues introduce how in lodgepole pines co-association networks can be used to study modularity and pleiotropic effects in multivariate climates [4].

Other studies focused on understanding the signals within genomes to illuminate demographic changes in the evolutionary history of species. Fernando Seixas, Pierre Boursot and José Melo-Ferreira examined the genomes of Iberian hares that were displaced from parts of their range but still hybridize, showing how selective effects determine which parts of the genomes are retained from each species [5]. Maja Mattle-Greminger and colleagues studied patterns of selection and admixture in the genomes of species of orangutan during their evolution on Sumatra and Borneo during the Pleistocene period [6]. The differences in ancient climate between the islands have left evidence of selection on different traits between the species inhabiting them to adapt to these specific challenges, and although the islands were on occasion joined when sea levels dropped, there is scant evidence of the species mixing.

Perhaps in no field of evolutionary biology has the synergy of new technical approaches and new sampling had so great an effect as in studies of our own species. The analysis of new archeological finds, new methods to reanalyze previously discovered samples, and the sampling of a broader pool of current human populations have revealed a complex picture of migration and admixture. In their study, Eugenia D’Atanasio and colleagues took samples from men from various parts of Africa to study the effects of the last greening of the Sahara, and this revealed a history of migration, pastoralism and evidence of a trans-Saharan slave trade [7]. Kristiina Tambets and colleagues showed evidence of the demographic and migratory events that underlie the spread of the Uralic languages from their origins in northern Siberia to as far to the West as Hungary [8].

In a review article, Mona Schreiber, Nils Stein and Martin Mascher reflect on how the development of genomic tools and datasets has expanded the study of the evolution of domesticated crop plants from their wild ancestors [9]. Initially, these efforts focused on the most widely cultivated and economically important crops. This review particularly notes that these approaches are increasingly being applied to the hundreds of more minor crops that have their own unique and enlightening histories of domestication.

Other studies in the series utilize the phylogenetic breadth of newly available data in comparative analyses to understand the evolution of the genome itself. Joana Damas and colleagues reconstructed the chromosomes of the ancestor of birds, finding that two of the very small microchromosomes, characteristic of birds, have been maintained intact over 100 million years [10]. Xavier Grau-Bové, Iñaki Ruiz-Trillo and Manuel Irimia looked at the early evolution of animals to discover the origins and expansion of a particular form of alternative splicing characteristic of animals and hypothesize why this is seen in animals, but not in other groups [11]. In another study, Jason Klein and colleagues show how the genome evolved across the primate evolutionary tree, finding patterns in their loss or gain and important mechanisms of how this occurs [12]. Genetic changes that affect how much a gene is expressed do not necessarily lead to equivalently large changes in the expression of the protein they code for. Also looking at primate genomes, Sidney Wang and colleagues find that a mechanism of post-translational gene expression buffering is conserved between species, making non-disruptive evolutionary changes more likely to occur [13]. Even further, evolutionary rate variation between genes can be explained by trade-offs in the complex optimization of codon usage, demonstrated in a study by Emily Seward and Steven Kelly [14].

Several of the studies bring insight into the evolution of developmental processes. Hua Ying and colleagues reported three new coral genomes, finding that uniquely among animals, robust corals are capable of de novo histidine biosynthesis [15]. Lauren Blake and colleagues described the development and validation of an early endoderm differentiation comparative genomics model in human and chimpanzee, confirming that gastrulation is a highly constrained variation and show that the process is evolutionarily conserved in humans and chimpanzees [16]. Wen-Juan Ma and colleagues study a population of frogs that are just developing a new sex chromosome, allowing them to test ideas about the relationship between genes expressed differently between the sexes [17]. Their findings challenge the idea that these accumulate on the new sex chromosomes and are central to their evolution.

As can be seen from the contributions to this special issue, the topics within evolutionary biology for which genomic data is being applied span the whole field. For some questions we are just scratching the surface of what remains to be discovered about the selective processes in play. New approaches will provide ever more, and more informative data, along with new challenges of how to analyze these, and genome manipulation methods give new possibilities to experimentally probe evolutionary hypotheses. It promises to be a thrilling time to be following the field.

Check the Collection page [1] to read these articles and more.
